# Pyomyositis in Northern California Following Provoked Deep Vein Thrombosis

**DOI:** 10.1155/crcc/8846829

**Published:** 2026-06-02

**Authors:** Nicholas Sparr, Avijit Baidwan, Nathan C. Claydon, Jessica McMichael, Glenn Gookin

**Affiliations:** ^1^ Medicine, Sierra Nevada Memorial Hospital, Grass Valley, California, USA; ^2^ Family Medicine, Sierra Nevada Family Medicine Residency Program, Grass Valley, California, USA; ^3^ Surgery, Sierra Nevada Memorial Hospital, Grass Valley, California, USA

## Abstract

Pyomyositis is an uncommon bacterial infection of the skeletal muscle, traditionally associated with tropical regions and immunocompromised hosts. We present a case of a 44‐year‐old immunocompetent female in Northern California who developed pyomyositis involving the vastus medialis, rectus femoris, and vastus lateralis. A drainable abscess was not initially demonstrated on computed tomography (CT) imaging, and diagnosis was delayed by findings of deep vein thrombosis (DVT) with septic pulmonary emboli in the setting of septic shock due to methicillin‐sensitive *Staphylococcus aureus* (MSSA) bacteremia. Two possible pathogenetic mechanisms were considered: either a provoked DVT from immobilization following recent lower extremity surgery served as a nidus for bacterial seeding, or alternatively, a superficial thigh abscess triggered a hypercoagulable state leading to DVT formation and subsequent bacterial seeding. In either scenario, muscle strain from impaired mobility rendered the tissues vulnerable to abscess formation. The patient required multiple surgical debridements and prolonged intravenous antibiotic therapy for successful treatment. This case reveals atypical pathogenetic mechanisms for pyomyositis and highlights the necessity for physicians to remain vigilant for this disease process that has demonstrated an increasing incidence rate in the United States, even in immunocompetent hosts and in temperate climates.

## 1. Introduction

Pyomyositis is a rare bacterial infection of the skeletal muscle. Historically, this condition has been predominantly observed in immunocompromised hosts and in tropical regions, earning the moniker “tropical pyomyositis” [1]. Its etiology is typically hematogenous spread from an occult focus [2], and *Staphylococcus aureus* is identified as the causative pathogen in 60%–90% of cases [3].

We report a case of a 44‐year‐old immunocompetent female residing in Northern California, United States, who presented with septic shock secondary to pyomyositis of the vastus medialis, rectus femoris, and vastus lateralis following muscle strain. A drainable abscess was not initially revealed on computed tomography (CT) imaging, and diagnosis was delayed by the initial finding of a nonocclusive common femoral vein deep vein thrombosis (DVT) in the affected extremity and septic pulmonary emboli. This case report will highlight an increasingly diagnosed disease in the United States that should remain on the differential of any patient presenting with extremity pain and sepsis.

## 2. Case

A 44‐year‐old female patient with a past medical history of hyperlipidemia, Class I obesity, and vitamin D insufficiency presented to the emergency department in July 2024 with 1 week of worsening left thigh pain, shortness of breath, and fever.

The patient’s clinical course began 59 days prior to hospital presentation when she fell on stadium bleachers, sustaining fractures to the fourth and fifth metatarsals of her right foot. Thirty‐five days before admission, she underwent open reduction and internal fixation of these fractures. Subsequently, 21 days prior to admission, she developed a left posterior thigh superficial abscess requiring incision and drainage at the office of her primary care physician. Two days after incision and drainage, she was prescribed cephalexin 500 milligrams (mg) four times daily for 5 days. Approximately 1 week before admission, she slipped and experienced acute pain in her left medial thigh. Her symptoms progressively worsened with fever, increasing thigh pain, and eventually dyspnea, prompting her to seek emergency care.

Initial laboratory studies revealed a white blood cell count of 10.8 K/*μ*L (reference range: 4.5–11.0 K/*μ*L), hemoglobin of 11.9 g/dL (12.0–16.0 g/dL), lactic acid of 3.6 mmol/L (0.5–2.0 mmol/L), procalcitonin of 15.14 ng/mL (< 0.5 ng/mL), and D‐dimer of 5748 ng/mL (< 500 ng/mL) (Table 1). Blood cultures were obtained, and lower extremity ultrasound revealed a nonocclusive left common femoral vein DVT. Chest CT demonstrated multiple peripheral nodular opacities consistent with septic pulmonary emboli.

**Table 1:** Presenting, peak, and trough key laboratory values during hospitalization.

Laboratory test	Reference range	At presentation	Peak value (Hospital Day)	Trough value (Hospital Day)
White blood cell (K/*μ*L)	4.5–11.0	10.8	27.3 (Day 7)	8.1 (Day 19)
Hemoglobin (g/dL)	12.0–16.0	11.9	11.9 (Day 0)	8.2 (Day 7)
Lactic acid (mmol/L)	0.5–2.0	3.6	4.5 (Day 2)	2.0 (Day 2)
Procalcitonin (ng/mL)	< 0.50	15.14	15.14 (Day 0)	0.15 (Day 11)
C‐reactive protein (mg/dL)	< 1.00	—	17.71 (Day 11)	4.63 (Day 15)
D‐dimer (ng/mL)	< 500	5748	—	—

The patient was admitted to the intensive care unit and started on intravenous (IV) unfractionated heparin (UFH) and broad‐spectrum antibiotics with ceftriaxone 1 g IV every 24 h and vancomycin dosed by trough level. Blood cultures subsequently grew methicillin‐sensitive *Staphylococcus aureus* (MSSA), and antibiotic therapy was narrowed to cefazolin 2 g IV every 8 h based on susceptibilities and the patient’s reported penicillin allergy. Despite appropriate antimicrobial therapy, she continued to experience worsening thigh pain, intermittent fevers, increasing oxygen requirements, and altered mental status.

Physical examination on Hospital Day 7 revealed exquisite tenderness of the left medial thigh with tense, warm overlying skin without crepitus or palpable fluctuance. The patient was unable to extend her left knee, and left hip strength was diminished. Laboratory studies at this time showed leukocytosis of 27.3 K/*μ*L (Table 1).

CT of the left lower extremity with contrast on Hospital Day 3 had revealed moderate cellulitis and edema within the fascial planes of the anterior and medial compartments of the proximal thigh (Figure 1), which were suggestive of fasciitis. Imaging was notable for heterogeneous enhancement of the vastus medialis at that time, which was compatible with myositis, but there was no discrete rim‐enhancing fluid collection.

**Figure 1:**
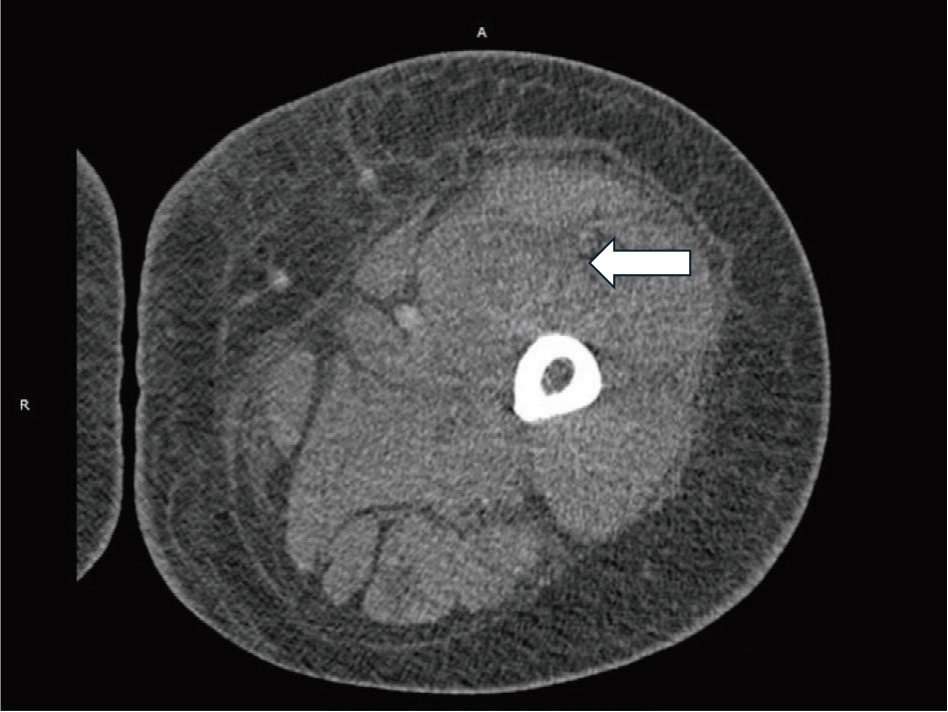
CT of the left lower extremity with contrast. Edema within the fascial planes of the anterior and medial compartments of the proximal thigh, suggestive of fasciitis. No soft tissue gas. Heterogeneous enhancement of the vastus medialis muscle compatible with myositis. No discrete rim‐enhancing fluid collection.

With worsening left medial thigh pain on Hospital Day 7, magnetic resonance imaging (MRI) with contrast was obtained for the left lower extremity, which demonstrated a fluid collection centered within the vastus medialis muscle measuring 7 × 5 cm in the axial dimension and 20 cm in the craniocaudal dimension (Figures 2 and 3). There was no osseous abnormality noted. Transthoracic echocardiogram and transesophageal echocardiogram were obtained during hospitalization to evaluate for endocarditis given findings of septic pulmonary emboli in the setting of MSSA bacteremia; however, no valvular vegetations or cardiac abnormalities were noted.

**Figure 2:**
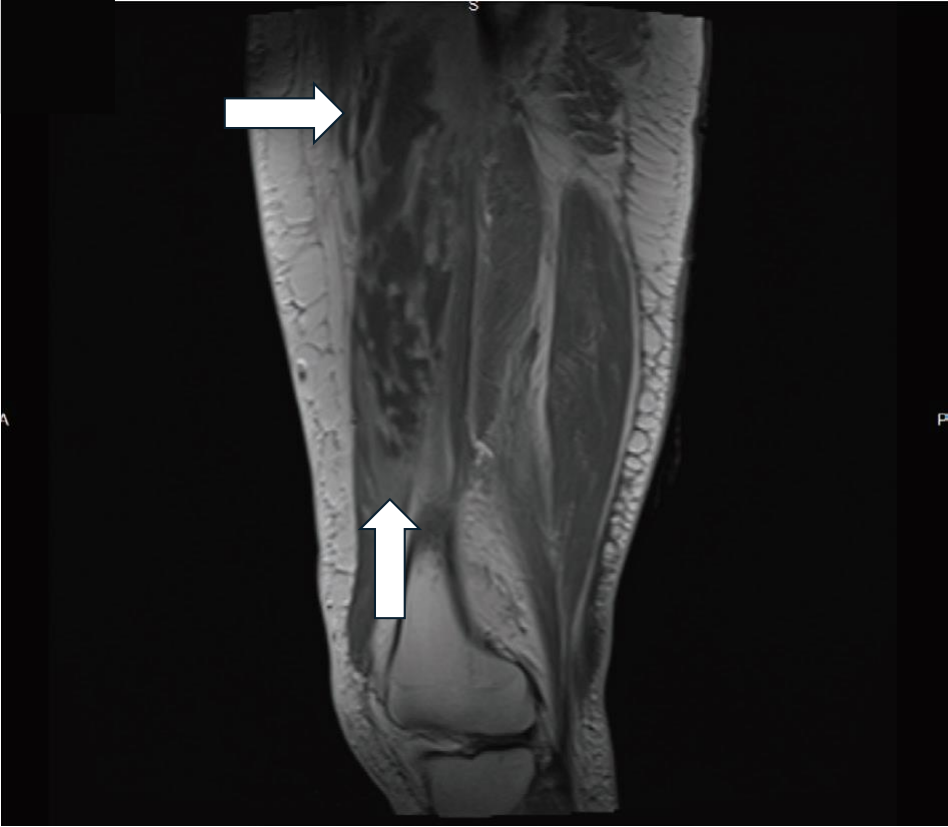
MRI of the left lower extremity with and without contrast demonstrating an irregular fluid collection in the deep anteromedial intramuscular compartment measuring 20 cm in the craniocaudal dimension.

**Figure 3:**
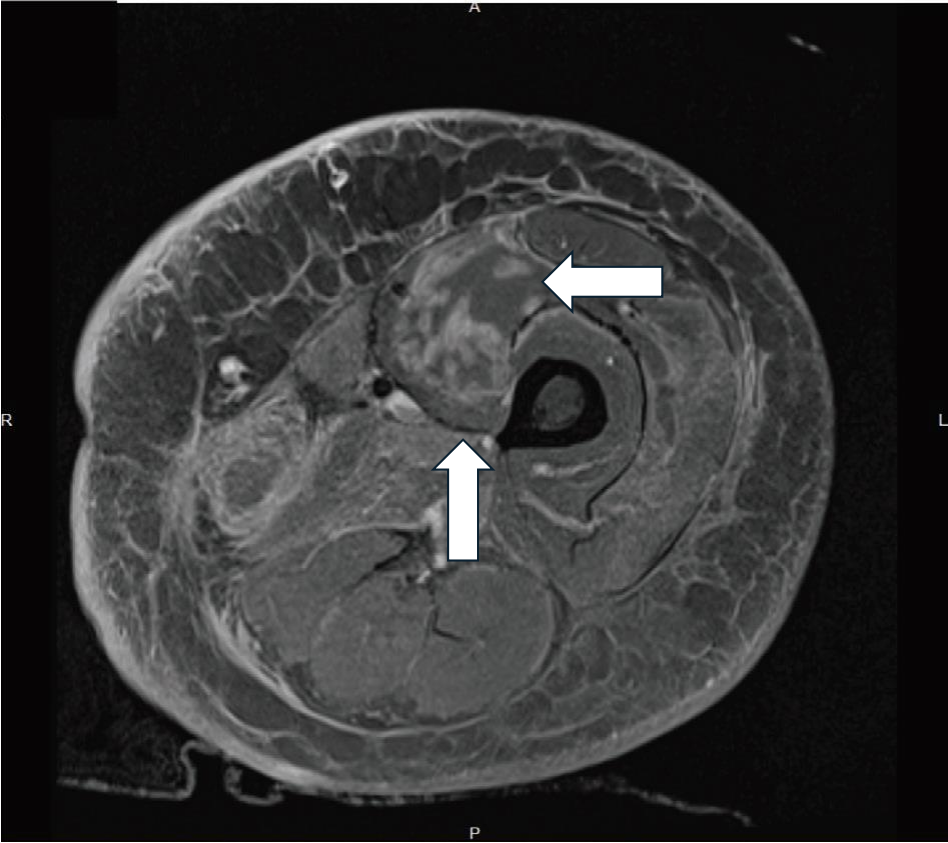
MRI of the left lower extremity with and without contrast demonstrating an irregular fluid collection in the deep anteromedial intramuscular compartment measuring 7 × 5 cm in the axial dimension.

The patient underwent three sequential surgical procedures for irrigation and debridement of the vastus medialis abscess (outlined in Table 2).

**Table 2:** Summarized timeline of events.

Hospital Day	Events
Day −59	**Initial trauma:** Fall on stadium bleachers resulting in right fourth and fifth metatarsal fractures.
Day −35	**Open reduction and internal fixation (ORIF):** Outpatient ORIF for right foot metatarsal fractures.
Days −21 to −19	**Left posterior thigh superficial abscess:** Outpatient incision and drainage of superficial left posterior thigh abscess, followed 2 days later by reevaluation with 5 days of cephalexin 200 milligrams (mg) four times daily.
Day −7	**Ground-level fall:** Patient fell while utilizing a scooter device following right foot metatarsal surgical intervention. The fall resulted in mild left medial thigh pain.
Day 0	**Presentation to the emergency department:** Patient admitted to the intensive care unit (ICU) for septic pulmonary emboli in the setting of left nonocclusive common femoral vein (CFV) deep vein thrombosis (DVT). Blood cultures grew methicillin‐sensitive *Staphylococcus aureus* (MSSA).
Day 3	**CT of left lower extremity:** Acute left medial thigh pain with CT imaging demonstrating cellulitis and fasciitis without a drainable pocket or abscess.
Day 7	**Worsening left medial thigh pain:** MRI with and without contrast revealed abscess formation consistent with pyomyositis. Orthopedic surgery was consulted, and incision and drainage (I&D) of the abscess was performed. Eliquis was discontinued on the day of the procedure, and I&D of the left thigh abscess in the deep anterior compartment was performed.
Day 11	**Repeat I&D:** Imaging demonstrated retained fluid in the left vastus intermedius and vastus lateralis. Repeat I&D followed by placement of a wound vacuum.
Day 13	**Deferral of third I&D:** The patient was to have a repeat I&D procedure, but the case was delayed due to an episode of hemoptysis that self‐resolved.
Day 15	**Final I&D:** Absence of fluid or necrotic tissue. The wound was debrided and irrigated. A wound vacuum was placed.
Day 19	**Patient discharged home:** Patient discharged to home with a wound vacuum and IV antibiotics for a total of 6 weeks of antibiotic therapy. Patient additionally discharged on oral apixaban.

Anticoagulation was managed initially with UFH for the first 48 h, followed by initiating apixaban 10 mg every 12 h for 7 days. With the first irrigation and debridement, the patient’s apixaban was discontinued approximately 12 h before the procedure. After the first debridement, it was apparent that the patient would need repeat procedures, and she was transitioned back to UFH to facilitate perioperative anticoagulation management. The UFH was discontinued 4 h prior to each subsequent irrigation and debridement and was resumed 12 h postoperatively. Intraoperative cultures confirmed MSSA infection.

Hypercoagulability workup revealed the presence of anticardiolipin antibody and heterozygosity for MTHFR c.665C>T, though the latter is likely not clinically significant. Following clinical improvement, she was discharged on Hospital Day 19 with a 6‐week course of nafcillin 2 g IV every 4 h. The patient was additionally discharged on apixaban 5 mg twice daily. At 6‐month follow‐up, repeat testing for anticardiolipin antibody was negative, suggesting against antiphospholipid syndrome as the cause of DVT.

## 3. Discussion

DVT is a recognized complication of pyomyositis due to direct venous compression by an adjacent muscular abscess [4, 5]. Pyomyositis with septic pulmonary emboli has been described in MRSA infections [6, 7], though also with MSSA [8]. This case is notable for the simultaneous occurrence of all three processes—pyomyositis, provoked DVT, and septic pulmonary emboli—in an immunocompetent host with MSSA infection. The pathogenetic mechanism in this case differs from prior reports: rather than DVT arising from local venous compression by an abscess, postsurgical immobilization, a preceding superficial abscess, and trauma‐induced muscle vulnerability suggest a complex sequence.

The diagnosis of pyomyositis was obscured by the concurrent finding of DVT with septic pulmonary emboli and the absence of leukocytosis. This diagnostic challenge mirrored previous cases in which pyomyositis mimicked or coexisted with DVT, delaying recognition [4, 5]. Despite appropriate treatment of MSSA bacteremia, persistent symptoms ultimately revealed a drainable abscess not identified on initial CT. Clinicians should maintain suspicion for deep musculoskeletal infection when patients fail to improve despite adequate antimicrobial therapy.

We propose two pathophysiological sequences. Decreased mobility after metatarsal fracture repair caused venous stasis, with transient bacteremia from the superficial thigh abscess seeding a preexisting DVT; muscle strain from a fall rendered the medial compartment vulnerable to abscess formation. Alternatively, the superficial abscess triggered a hypercoagulable state through tissue factor–mediated thrombin generation and impaired fibrinolysis [9], precipitating DVT that was seeded by bacteremia. Neither mechanism involves venous compression by an adjacent abscess that has characterized prior reports [5].

Prior reports of pyomyositis with septic pulmonary emboli have predominantly involved MRSA [6, 7, 10] without concurrent DVT [6–8, 10]. This case differed in that MSSA was the causative organism, a provoked DVT was present as a possible nidus for bacterial seeding, and a superficial abscess was the identifiable source of bacteremia after endocarditis was excluded. This case reinforces the limitation of CT in early pyomyositis; the initial CT identified cellulitis and myositis but no drainable collection, consistent with published cases [11]. MRI revealed a 7 × 5 cm fluid collection within the vastus medialis extending 20 cm craniocaudally, enabling definitive surgical planning. When clinical suspicion for musculoskeletal infection remains high despite unremarkable CT findings, MRI should be the preferred modality [12].

With pyomyositis increasing in incidence in temperate regions among immunocompetent individuals [13, 14], this case illustrates an atypical pathogenetic mechanism in which provoked DVT, transient bacteremia, and trauma‐induced muscle vulnerability converged to produce pyomyositis complicated by septic pulmonary emboli. Clinicians should maintain a high index of suspicion for pyomyositis in patients with persistent extremity pain and systemic inflammation that fail to resolve with appropriate antimicrobial therapy.

## Funding

No funding was received for this manuscript.

## Conflicts of Interest

The authors declare no conflicts of interest.

## Data Availability

Data sharing is expected.
